# The complete mitochondrial genome of *Eumeta variegata* (Lepidoptera: Psychidae)

**DOI:** 10.1080/23802359.2018.1495119

**Published:** 2018-08-01

**Authors:** Kazuharu Arakawa, Nobuaki Kono, Rintaro Ohtoshi, Hiroyuki Nakamura, Masaru Tomita

**Affiliations:** a Institute for Advanced Biosciences, Keio University, Tsuruoka, Japan;; b Faculty of Environment and Information Studies, Keio University, Fujisawa, Japan;; c Spiber Inc., Tsuruoka, Japan

**Keywords:** Mitochondrial genome, *Eumeta variegata*, *Eumeta japonica*, bagworm moth, nanopore sequencing

## Abstract

The complete mitochondrial genome of *Eumeta variegate*, largest bagworm moth in Japan, has been sequenced using a nanopore sequencer as a single long read. The genome has a total length of 16,601 bp, consisting of 13 protein-coding genes, 20 tRNA, 2 rRNA genes, and an AT-rich control region. The nucleotide composition was extremely AT-rich, with 42.4% A, 40.4% T, 6.67% G, and 10.6% C. This is the second report of a complete mitochondrial genome of Psychidae, and the sequence information together with a phylogenetic analysis would provide a reference data in the future studies of Lepidoptera and Psychidae.

The bagworm family (Leidoptera: Psychidae) is comprised of approximately 1000 species and 300 genera (Rhainds et al. 2009). The genus *Eumeta* is widely distributed among Asia-Pacific and Africa, but its taxonomy remains relatively less well curated due to the high morphological variation. The largest bagworm in Japan, *E. japonica* have recently been revised to be a synonym of *E. variegata* (Ong 2009), and the availability of complete mitochondrial genomes from this species provides a reference in the phylogenetic study of *Eumeta*. Moreover, the exceptional physical properties of the silk, these bagworms use to form their bag and attach themselves onto a substrate are also being researched (Wolff et al. 2017), especially with considerations for possible industrial applications.

The last instar larva was collected from Chiba, Japan (35.813278, 140.403883). The specimen is stored in the Institute for Advanced Biosciences, Keio University, Japan accession number: IMPACT-IDV02947A. High molecular weight DNA was extracted using Genomic-tip 20/G (QIAGEN) and was sequenced using 1D ligation sequencing kit and R9.4 flow cell on GridION X5 system (Oxford Nanopore Technologies). Longest read matching to mitochondrial genes was selected using BLAST searches (Altschul et al. 1997), and the read was subsequently error corrected with nanopolish software (Simpson et al. 2017) and further corrected using Illumina reads using Pilon (Walker et al. 2014) and proovread (Hackl et al. 2014). Circularity was checked manually, and the genome was annotated using MITOS2 WebServer (Bernt et al. 2013).

The complete mitochondrial genome sequence of *Eumeta variegata* has a total length of 16,601 bp DDBJ accession number AP018693), consisting of 13 protein-coding genes, 20 tRNA, 2 rRNA genes, and an AT-rich control region. Gene order and sequences are highly conserved in comparison to previously described complete genome of another Psychidae, *Mahasena colona.* Phylogenetic analysis (Figure 1) confirms this relatively close distance of the two genera of Psychidae in comparison to other Lepidoptera genera.

**Figure 1.**
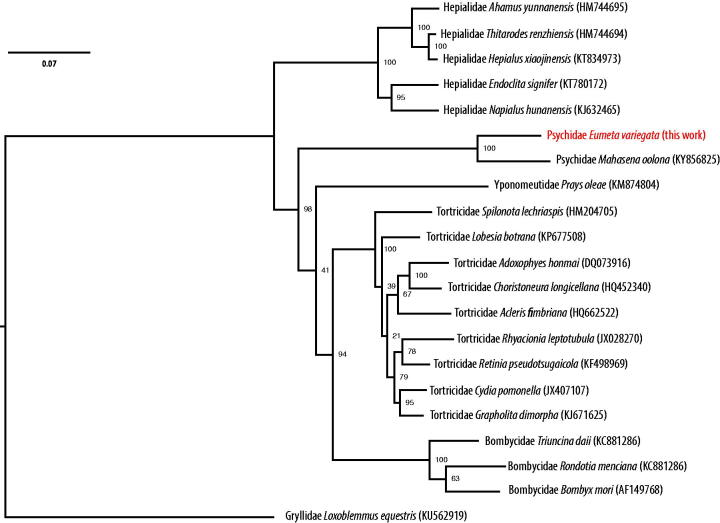
A maximum-likelihood tree of the phylogenetic position of *E. variegata* among other Leidoptera species. The tree was calculated from concatenated amino acid sequences of 13 mitochondrial protein genes using multiple alignment with MAFFT (Katoh and Standley 2014), followed by Trimal (80% consensus) and RAxML (Stamatakis 2015) with 100 bootstraps. Tree is visualized with FigTree (http://tree.bio.ed.ac.uk/software/figtree/). Orthoptera was used as an outgroup. GenBank accession numbers of mitogenome sequences used is shown in parentheses.
